# Academic Life in Emergency Medicine (ALiEM) Blog and Podcast Watch: Infectious Diseases

**DOI:** 10.7759/cureus.7674

**Published:** 2020-04-15

**Authors:** David Ediger, Ryan Sumpter, Rachel E Bridwell, Christopher N Belcher

**Affiliations:** 1 Department of Military and Emergency Medicine, Uniformed Services University of the Health Sciences, F. Edward Hébert School of Medicine, Bethesda, USA; 2 Department of Emergency Medicine, Brooke Army Medical Center, Fort Sam Houston, USA

**Keywords:** emergency medicine, free open access medical education, sepsis, fournier gangrene, infectious diseases, toxic shock syndrome, cellulitis

## Abstract

The Academic Life in Emergency Medicine (ALiEM) Approved Instructional Resources (AIR) Series and Approved Instructional Resources - Professional (AIR-Pro) Series were created in 2014 and 2015, respectively, in response to the growing need to curate online educational content as well as create a nationally available curriculum that meets individualized interactive instruction criteria for emergency medicine (EM) trainees. These two online series identify high-quality educational blog and podcast content using an expert-based approach.

The AIR series is a continuously building curriculum originally based on the Council of Emergency Medicine Directors (CORD) testing schedule. In September 2019, 61 blog posts and podcasts published within the previous 12 months and relevant to infectious diseases were evaluated by eight attending physicians using the ALiEM AIR scoring instrument. In this review, we summarize the accredited posts on infectious diseases meeting our a priori quality criteria per evaluation by the reviewers.

## Introduction and background

Emergency medicine (EM) has been at the forefront in the adoption of social media platforms as a means to disseminate knowledge with a rapid rise of educational content available through blogs and podcasts [1]. However, standardized assessments of the quality of these resources have only recently begun to be developed [2-4]. Additionally, in 2008, the Accreditation Council for Graduate Medical Education allowed EM residency programs to move some of their in-person conference experience to individualized learning, termed Individualized Interactive Instruction (III) [5]. This established a need for a national curriculum with a standardized process for assessing the quality of online educational resources while also meeting requirements to qualify for III. The Academic Life in Emergency Medicine (ALiEM) Approved Instructional Resources (AIR) Series and Approved Instructional Resources - Professional (AIR-Pro) Series were created in 2014 and 2015, respectively, to fill these gaps [6-7]. These two programs identify high-quality educational blog and podcast content. The ALiEM Blog and Podcast Watch series is authored by members of the AIR and AIR-Pro Series’ editorial boards to create written summaries of these selected posts [8-17]. This installment from the series summarizes the highest-scoring social media educational resources on infectious diseases as determined by expert opinion.

## Review

Topic identification

The AIR Series is a continuously building curriculum structured to cover all relevant organ systems.

Inclusion and exclusion criteria

A search of the 50 most frequently visited sites per the Social Media Index was conducted for resources relevant to infectious diseases published within the previous 12 months [18-19]. The search, conducted in September 2019, included blog posts and podcasts written in English for scoring by our expert panel.

Scoring

Extracted posts were scored without blinding by eight reviewers from the AIR editorial board, which is constituted of EM core faculty from various United States medical institutions. The scoring instrument contains five measurement outcomes using seven-point scales: Best Evidence in Emergency Medicine (BEEM) score, accuracy, educational utility, evidence-based medicine, and references (Table 1) [20]. More detailed methods are described in the original description of the AIR Series [6-7]. Board members with any role in the production of a reviewed resource recuse him/herself from grading that resource.

**Table 1:** Approved Instructional Resources scoring instrument for blogs and podcasts BEEM: Best evidence in emergency medicine; EP: Emergency physician; EBM: Evidence-based medicine

Tier 1: BEEM Rater Scale	Score	Tier 2: Content Accuracy	Score	Tier 3: Educational Utility	Score	Tier 4: Evidence-Based Medicine	Score	Tier 5: Referenced	Score
Assuming that the results of this article are valid, how much does this article impact on EM clinical practice?		Do you have any concerns about the accuracy of the data presented or the conclusions of this article?		Are there useful educational pearls in this article for senior residents?		Does this article reflect evidence-based medicine (EBM)?		Are the authors and literature clearly cited?	
Useless information	1	Yes, many concerns from many inaccuracies	1	Not required knowledge for a competent EP	1	Not EBM based, only expert opinion	1	No	1
Not really interesting, not really new, changes nothing	2		2		2		2		2
Interesting and new, but doesn't change practice	3	Yes, a major concern about few inaccuracies	3	Yes, but there are only a few (1-2) educational pearls that will make the EP a better practitioner to know or multiple (>=3) educational pearls that are interesting or potentially useful, but rarely required or helpful for the daily practice of an EP.	3	Minimally EBM based	3		3
Interesting and new, has the potential to change practice	4		4		4		4	Yes, authors and general references are listed (but no in-line references)	4
New and important: this would probably change practice for some EPs	5	Minimal concerns over minor inaccuracies	5	Yes, there are several (>=3) educational pearls that will make the EP a better practitioner to know, or a few (1-2) every competent EP must know in their practice	5	Mostly EBM based	5		5
New and important: this would change practice for most EPs	6		6		6		6		6
This is a "must-know" for EPs	7	No concerns over inaccuracies	7	Yes, there are multiple educational pearls that every competent EP must know in their practice	7	Yes, exclusively EBM based	7	Yes, authors and in-line references are provided	7

Resources with a mean evaluator score of ≥ 30 points (out of a maximum of 35) are awarded the AIR label. Resources with a mean score of 27-29 and deemed accurate and educationally valuable by the reviewers are given the Honorable Mention (HM) label.

Results

We initially evaluated 61 posts on the topic of infectious diseases from the top 50 websites meeting our search criteria, from which we identified one AIR and eight honorable mentions. Key educational pearls from these resources are described below.

Article 1 (AIR): Helman, A. Gray, S., Morgenstern, J., Spiegel R., Kovacs, G., Simard, R. Sepsis and Septic Shock - What Matters. Emergency Medicine Cases. March 2019.

https://emergencymedicinecases.com/sepsis-septic-shock. Accessed March 20, 2020

Take-home points: this blog post/podcast reviews the latest evidence and provides an expert opinion on the recognition and management of sepsis and septic shock.

Summary

The diagnosis of sepsis anchors on suspected infection with underlying organ dysfunction. In the myriad of scoring systems, the NEWS score is the most accurate clinical tool in identifying the clinically ill early within the sepsis cohort and consists of the following items: level of awareness, temperature, systolic blood pressure, respiratory rate, supplemental oxygen requirement, and heart rate. Lactate may be helpful when the diagnosis is unclear but should not be used as a resuscitation endpoint and can generate false positives. Procalcitonin is not useful for identifying occult sepsis nor guiding resuscitation. Early administration of antibiotics decreases mortality; administer the antibiotic with the greatest coverage against the suspected source first and consider oral administration if there is high bioavailability. Empiric antibiotics should be dictated by local antibiograms to minimize resistance.

Ringer’s Lactate (RL) is the preferred resuscitation fluid though it may require a second IV line due to incompatibility with medications. Normal saline is less ideal given it can cause metabolic acidosis and renal vasoconstriction from the chloride load. Patients should receive crystalloid boluses and fluid resuscitation should be titrated to maintain end-organ perfusion. In patients with hypotension (mean arterial pressure (MAP) <65) refractory to fluid resuscitation, consider norepinephrine early -- through a large, well-placed peripheral line if necessary. Vasopressin should be considered if MAP remains <65 on norepinephrine though before norepinephrine has reached its maximum useful dose. Steroids should be reserved for patients with vasopressor-refractory shock, chronic steroid use, or adrenal suppression.

Article 2 (honorable mention, HM): Weingart S. EMCrit Podcast 241 - Sepsis Update 2019. EMCrit Blog. Published on February 21, 2019.

https://emcrit.org/emcrit/emcrit-podcast-241-sepsis-update-2019/

Take-home points: this podcast discussed three new studies on sepsis management: ANDROMEDA-SHOCK, CENSER, and HiTEMP. It examines the goals of resuscitation in sepsis, the early use of low-dose norepinephrine in sepsis for fluid conservative management, as well as another trial that does not find utility in procalcitonin to differentiate bacterial from non-bacterial infections.

Summary

Using ideal body weight rather than actual body weight may mitigate excess fluid in septic patients while abiding by a 30mL/kg crystalloid bolus policy. Clinicians should be sure to document their rationale if performing this. Medication infusion volumes given within the time window also contribute to this total volume goal.

The ANDROMEDA-SHOCK trial randomized septic patients with a lactate >2 and on vasopressors to resuscitation protocols guided by lactate clearance (20% decrease in two hours) or normalizing capillary refill time (CRT; <3 seconds). Patients not meeting goals based on their randomized group were given fluid boluses, followed by vasopressors, and then inodilators. The CRT arm had an 8.5% lower mortality (all-cause at 28 days) than the lactate arm and received less fluid overall. Meanwhile, the lactate clearance group had a notable trend towards increased mortality. While the results were not statistically significant, this clinical trend indicates markers such as capillary refill may be useful as a guide for resuscitation.

The prospective randomized CENSER trial studied low-dose norepinephrine in patients with septic shock. A fixed-dose peripheral norepinephrine drip (0.05 mcg/kg/min) resulted in resolution of hypotension (MAP >65) and oliguria (UOP >0.5 mL/kg/hr) compared to usual sepsis care. Though not statistically significant, mortality was lower in the norepinephrine group. Low-dose norepinephrine increases venous constriction, possibly resulting in more blood volume returning to the heart. Additionally, fixed-dose norepinephrine given through a well placed peripheral vein may allow dispositioning of patients to a non-ICU level of care.

The HiTEMP trial showed that in patients presenting to the Emergency Department with fever, procalcitonin does not reliably differentiate bacterial infection from other causes of fever. Procalcitonin is not yet ready for ED application for the differentiation of bacterial versus viral infections.

Article 3 (HM): Woods J. New PECARN Febrile Infant Rule: A 3-Variable Approach for Ages 29-60 Days. ALiEM. June 19, 2019.

https://www.aliem.com/pecarn-infant-fever-rule-age-29-60-days/

Take-home points: this post/podcast reviewed a new clinical prediction rule for identifying febrile infants between 29 and 60 days at low risk for serious bacterial infection (SBI) using urinalysis (UA), absolute neutrophil count, and procalcitonin.

Summary

Febrile infants often undergo a battery of testing and hospitalization to rule out SBI (urinary tract infection, meningitis, bacteremia). This one-directional, progressive rule uses urinalysis, absolute neutrophil count, and procalcitonin to identify otherwise healthy febrile infants under 60 days who are at low risk for a serious bacterial infection. A positive urinalysis was defined as the presence of leukocyte esterase, nitrites, >5 WBC/hpf, or trace bacteria. Based on a cohort of 1,896 patients, having a negative UA, absolute neutrophil count (ANC) ≤ 4,090/mL, and procalcitonin ≤ 1.71 ng/mL (sensitivity 98.8%, specificity 63.31%) places a febrile infant into the low-risk category for SBI and may not require lumbar puncture, antibiotics, or admission. This rule supplements but does not replace clinical judgment and should be used with caution in infants under 28 days out of concern for herpes encephalitis.

Article 4 (HM): Rezaie S. The Tamiflu Debacle. REBEL EM blog. October 24, 2018.

https://rebelem.com/the-tamiflu-debacle/

Take-home points: this post examines three systematic reviews on the efficacy and safety of oseltamivir (Tamiflu).

Summary

Oseltamivir is a neuraminidase inhibitor with Food and Drug Administration (FDA) approval for the treatment and prophylaxis of influenza A and B. Three recent systematic reviews analyzed available evidence for oseltamivir’s effect on time to alleviation of flu symptoms, direct transmission of influenza, and rates of symptoms, hospitalization, and antibiotic use [21-23]. Oseltamivir reduces flu-like symptoms in adults by about 12 hours and non-asthmatic children by 24 hours, although it does not reduce rates of hospitalization or complications. There is also an increased risk of nausea, vomiting, headache, and neuropsychiatric illness. It appears to prevent person-to-person and household transmission of the flu, though it is unclear whether it prevents community transmission. Based on the available evidence, oseltamivir should not be recommended for immunocompetent and otherwise healthy adults or children for influenza treatment or prophylaxis. Healthcare providers should engage in shared decision-making with patients regarding the benefits, harms, and costs of oseltamivir.

Article 5 (HM): Montrief T, Auerbach J, Koyfman A, Long B. Fournier’s Gangrene: ED presentations, evaluation, and management. emDOCs. July 1, 2019.

http://www.emdocs.net/fourniers-gangrene-ed-presentations-evaluation-and-management/

Take-home points: Fournier gangrene (FG) most commonly presents in obese older males who are immunosuppressed, diabetic, or have alcohol use disorder. They are most commonly polymicrobial and 75% of cases are initially diagnosed as cellulitis. Providers should look for ecchymosis, pain out of proportion, or crepitus. Empiric broad-spectrum antibiotics and emergent surgical debridement are the mainstays of treatment.

Summary

FG is a rare but serious necrotizing soft tissue infection (NSTI) extending into the perineal, perianal, and genital area. Incidence has been reported at 1.6 per 100,000 men with a peak between the ages of 50 and 79 years (3.3 per 100,000) and an incidence of 0.25 per 100,000 in women. While rare, overall case fatality can range from 7.5% to upwards of 50% [24]. Risk factors for developing FG include diabetes mellitus, obesity, hypertension, human immunodeficiency virus (HIV) infection, CKD, PVD, immunocompromise, and alcohol or tobacco use. 

FG is rapid, with the necrotizing process spreading along the fascial planes at a rate of up to 2-3 cm/hour [25]. Part of the reason for this rapid spread is due to the anatomy of the fascial planes of the perineum and deep fascial planes of the genitalia. Specifically, the Colles fascia remains continuous with other surrounding fascial planes, facilitating rapid spread towards the abdomen and thorax (via Scarpa’s fascia), as well as the scrotum (via Buck’s and Dartos fascia). The most common sources of FG arise from the gastrointestinal (GI) tract, genitourinary (GU) tract, and cutaneous injuries, in that order.

FG is a clinical diagnosis and often misdiagnosed as cellulitis or abscess in 75% of cases. Clinical features include tachycardia, fever, and the affected area appearing swollen, dusky, and with a possible purulent “dishwater” discharge. Pain out of proportion to the physical exam should alert the physician to possible FG. The extent of necrosis is one of the most important prognostic factors, and studies have shown that cases with necrotic areas of less than 3% of the total body surface area are rarely fatal [26]. The Laboratory Risk Indicator for Necrotizing Fasciitis (LRINEC) may help suggest the presence of NSTI but is not sensitive or specific enough for all cases. Radiographs may show evidence of gas formation in nearly half of FG patients and are highly specific (94%) though not sensitive [27]. While a point-of-care (POC) ultrasound can show “dirty shadowing,” its insufficient sensitivity (88%) does not allow for it to be a primary imaging modality. CT has a similar sensitivity (88.5%) though it is highly specific (93.3%) and can distinguish fascial planes as well as elucidate fistulae, abscesses, or other infectious processes [28]. While MRI is best at imaging soft tissues, its cost and time-consuming nature preclude its clinical utility for FG.

Management consists of emergent surgical debridement of all necrotic tissue, empiric broad-spectrum antibiotics, and hemodynamic resuscitation with intravenous (IV) fluids and vasoactive medications as needed. Delays in surgical debridement are directly related to the mortality of FG. A carbapenem plus clindamycin plus vancomycin/daptomycin should be initiated. Doxycycline should be added if saltwater or freshwater exposure is suspected, to cover Aeromonas species. Those at risk for a fungal disease should be treated with amphotericin B. Because of the significant comorbidity of alcohol use disorder in the FG risk population, emergency clinicians should have a low threshold for investigating alcohol withdrawal or delirium tremens, occurring in up to 50% of FG patients [29].

Article 6 (HM): Laurence C, Modi S, Hocker E. Deep Dive: Cellulitis Antibiotics Review. Taming the SRU. July 8, 2019.

http://www.tamingthesru.com/blog/minor-care-series/deep-dive-cellulitis-antibiotics-review

Take-home points: this article reviewed current best practices for antibiotic therapy selection for covering non-purulent cellulitis, how to best make the diagnosis of non-purulent cellulitis, and special considerations for methicillin-resistant Staphylococcus (S.) aureus (MRSA).

Summary

Cellulitis is a clinical diagnosis, but the first distinction that should be made is between purulent and non-purulent cellulitis. This can best be accomplished using point-of-care ultrasound. This modality has been found to have a sensitivity of 96.2% and a specificity of 82.9% in differentiating an abscess versus cellulitis in soft tissue infections. Further classification of non-purulent cellulitis to assist in determining antibiotic choice include mild (no evidence of systemic signs or symptoms of infection, and well-controlled or no comorbidities), moderate (evidence of systemic signs or symptoms of infection, poorly controlled comorbidities), or severe (presence of sepsis with altered mentation, toxic appearance, hypotension, end-organ dysfunction).

Based on the above clinical diagnosis, mild non-purulent cellulitis can be treated with an oral antibiotic, such as cephalexin, penicillin VK, dicloxacillin, or clindamycin (for those with penicillin allergy). Clinicians should consider intravenous antibiotics for moderate infections, including cefazolin, ceftriaxone, nafcillin/oxacillin, or clindamycin (for those with penicillin allergy). Severe infections should be treated with intravenous antibiotics such as vancomycin +/- piperacillin/tazobactam, meropenem/imipenem, or linezolid. 

Consider MRSA in the presence of the following risk factors: cellulitis with penetrating trauma, MRSA nasal colonization, concurrent or past history of MRSA infection, intravenous drug use, immunocompromised, and failed initial treatment. Oral antibiotic coverage for MRSA includes trimethoprim-sulfamethoxazole (TMP-SMX), clindamycin, doxycycline, and linezolid. Intravenous antibiotic coverage for MRSA includes vancomycin, TMP-SMX, clindamycin, and linezolid.

Article 7 (HM): Pulvino C, Li J, Hooker E. Antibiotic Review: Sexually Transmitted Infections. Taming the SRU. August 13, 2019.

http://www.tamingthesru.com/blog/minor-care-series/antibiotic-review-sexually-transmitted-infections

Take-home points: this article summarizes the most common STIs that present to the ED, as well as the proper treatment regimen for each. It also covers important areas of management such as reported sexual assault, post-exposure prophylaxis, and antibiotic resistance. 

Summary

Current Center for Disease Control and Prevention (CDC) guidelines recommend point-of-care diagnostic testing and subsequent treatment for suspected gonorrhea/chlamydia (GC) presenting to the ED. If POC testing is not available, patients should be treated empirically for GC while awaiting lab results. The currently recommended treatment regimen includes ceftriaxone and azithromycin, or gentamicin and azithromycin (for cephalosporin allergies), or ceftriaxone and doxycycline (for azithromycin allergies).

History and physical exams are essential tools for the diagnosis of sexually transmitted infection (STI) along with assessing for more severe signs of pelvic inflammatory disease (PID) or tubo-ovarian abscess (TOA). Testing for HIV and syphilis should be considered for patients with risk factors (multiple partners, concurrent drug use, multiple STIs) as well as rectal and oropharyngeal testing. In the setting of suspected or confirmed sexual assault, the CDC recommends empiric treatment for chlamydia, gonorrhea, and trichomonas, hepatitis B vaccination, and the option for emergency contraception. Post-exposure prophylaxis for HIV exposures can be initiated on an individualized basis though it should not be started if the time since exposure is greater than 72 hours. This article also covered specific treatment regimens for trichomoniasis, epididymitis, genital herpes simplex virus (HSV), syphilis, lymphogranuloma venereum, and pediculosis pubis.

Article 8 (HM): Fox S. Toxic Shock Syndrome. Ped EM Morsels. March 1, 2019.

https://pedemmorsels.com/toxic-shock-syndrome/

Take-home points: this article reviews toxic shock syndrome (TSS) in reference to pediatric patients. This challenging diagnosis is based on clinical criteria, which is caused by exotoxin release by Staphylococcusand Streptococcusspecies. Treatment is similar to the usual sepsis care with the important inclusion of clindamycin to block exotoxin effects and source discovery/control. Clinicians should maintain a high index of suspicion for TSS and a low threshold to initiate treatment.

Summary

TSS is a severe, acute illness, which may begin with non-specific symptoms and subsequently rapidly deteriorate. It is manifested in pediatric patients by high fever, hypotension, rash, multiorgan dysfunction, and desquamation. These characteristics can be transient, delayed, or altogether absent, prolonging diagnosis and initiation of treatment. 

TSS is caused by potent superantigen exotoxins, most commonly produced by Staphylococcus aureus and Streptococcus pyogenes. This is seen more commonly in patients under two years old and those with recent viral infections, congenital heart disease, and immune suppression. These exotoxins directly stimulate T-cells, leading to massive polyclonal cell proliferation. *S. aureus* occurs more frequently in older, female patients associated with menstruation (due to tampon usage), while streptococcal TSS is less common overall but more clinically severe, and more common, in younger children. In critically ill children presenting with erythroderma, conjunctival hyperemia, strawberry tongue, and a known nidus of infection (e.g., tampon use, nasal wound packing), the differential diagnosis should include TSS, as well as septic shock, Kawasaki disease, and drug reaction with eosinophilia and systemic symptoms (DRESS) syndrome. 

Management includes recognition of shock, rapid diagnostic testing, aggressive volume resuscitation, and initiation of broad-spectrum antibiotics (including clindamycin), ideally within the first 60 minutes. Additionally, management hinges upon source control, which may include the removal of foreign bodies or surgical drainage of an abscess or deep space infection. Antibiotics like clindamycin, rifampin, or linezolid may interfere with exotoxin production and release; clindamycin should be added to broad-spectrum coverage when TSS is presumed or confirmed.

Article 9 (HM): Fox S. Tracheitis in Children: a 2018 Update. Ped EM Morsels. November 28, 2018.

https://pedemmorsels.com/tracheitis-in-children-a-2018-update/

Take home points: this article reviews tracheitis in pediatric patients. Clinicians should consider tracheitis for children presenting with severe croup, recurrent croup, or croup that is not responding to the usual treatment. This can be an emergency due to airway compromise. It is most commonly caused by S. aureus. Treatment involves controlling the airway and antibiotics.

Summary

Tracheitis is an inflammation of the trachea (by infection or irritation), usually in combination with inflammation of nearby structures such as the larynx and bronchus. Croup, or viral laryngotracheobronchitis, is a common entity in childhood. However, clinicians treating presumptive croup should also consider bacterial tracheitis, which can quickly progress to life-threatening airway compromise. The most common cause of bacterial tracheitis is methicillin-sensitive S. aureus and Haemophilus influenzae in unvaccinated children. Viral coinfection is common. Bacterial tracheitis presents on a spectrum from mild to toxic-appearing and should be considered in any child presenting with cough, stridor, respiratory distress, fever, hoarse voice, orthopnea, or dysphagia. Clinicians should maintain a high index of suspicion for bacterial tracheitis when children have recurrent or severe croup or do not respond to the usual croup treatments such as steroids and racemic epinephrine. Plain films of the chest or neck may show evidence of tracheitis but are not useful in ruling out disease or predicting decompensation. Specialists should be engaged early for flexible endoscopy or bronchoscopy to aid in the diagnosis and establishment of a definitive airway. Management also includes resuscitation with fluids and vasopressors, as needed, and antibiotics to cover the most common pathogens.

## Conclusions

The ALiEM Blog and Podcast Watch series identifies high-quality educational blogs and podcasts for EM clinicians through its expert panel, using an objective scoring instrument. These social media resources are currently curated in the ALiEM AIR Series, originally created to address EM residency needs. While this article focuses on infectious disease emergencies, additional AIR modules address other topics in EM. The resources chosen specifically for infectious diseases are shared and summarized here, to help clinicians and educators filter through the rapidly published multitude of blog posts and podcasts. Our search was limited to content produced within the previous 12 months from the top 50 Social Media Index sites. While these lists are by no means a comprehensive analysis of the entire Internet for this topic, the AIR and AIR-Pro Series provide post-publication accreditation and curation of recent online content to identify and recommend high-quality, educational, social media content for the EM clinician.
